# Thyroid autoimmunity in Greenlandic Inuit

**DOI:** 10.1530/ETJ-22-0071

**Published:** 2022-04-28

**Authors:** Paneeraq Noahsen, Karsten F Rex, Inge Bülow Pedersen, Gert Mulvad, Hans Christian Florian-Sørensen, Michael Lynge Pedersen, Stig Andersen

**Affiliations:** 1 Arctic Health Research Centre, Department of Clinical Medicine, Aalborg University, Aalborg, Denmark; 2 Ilisimatusarfik, University of Greenland, Nuuk, Greenland; 3 National Board of Health, Nuuk, Greenland; 4 Department of Internal Medicine, Queen Ingrid’s Hospital, Nuuk, Greenland; 5 Department of Endocrinology, Aalborg University Hospital, Aalborg, Denmark; 6 Queen Ingrid’s Health Care Centre, Nuuk, Greenland; 7 Tasiilaq Health Care Center, Tasiilaq, Greenland; 8 Steno Diabetes Center Nuuk, Nuuk, Greenland; 9 Department of Geriatric Medicine, Aalborg University Hospital, Aalborg, Denmark

**Keywords:** thyroid autoimmunity, iodine excretion, Greenland Inuit, Arctic environment

## Abstract

**Objective:**

This study aimed to provide the first data on the occurrence of thyroid autoimmunity among Inuit in Greenland, a distinct ethnic group who is not iodine deficient.

**Design:**

This study is a population-based cross-sectional study.

**Methods:**

Data were collected in Nuuk in West Greenland and in Ammassalik district in East Greenland. Information on lifestyle, diet and diseases was obtained using questionnaires. Thyroid peroxidase antibody (TPOAb), thyroglobulin antibody (TGAb) and thyroid-stimulating hormone (TSH) were measured in serum. Iodine and creatinine were measured in spot urine samples.

**Results:**

The participation rate was 95% with 434 Inuit participants; 75% were smokers. Iodine excretion was 169 µg/24 h in urban West Greenland, 224 µg/24 h in the main town and 228 µg/24 h in settlements in rural East Greenland. TPOAb, TgAb or either of these was measured in the serum from 3.7, 5.9 and 8.3% of participants, respectively. TPOAb or TgAb was found in 9.3% of Inuit women and 7.5% of men and more frequently, in East Greenland Inuit with the higher iodine excretion (*P*  = 0.02). There was some evidence suggesting that thyroid autoimmunity was more frequent among non-smokers (12.5%) compared to smokers (7.0%). Harbouring a thyroid autoantibody was most frequent in participants with TSH above 3.6 mIU/L (*P*  < 0.001).

**Conclusion:**

Thyroid autoantibodies were rare among Greenland Inuit. While iodine nutrition was associated with autoimmunity similarly to other ethnic groups, the influence of sex and smoking was limited. This could suggest genetic component in Inuit, but the impact of cold, selenium and persistent organic pollutants needs to be elucidated.

## Introduction

Iodine intake level is important for the occurrence of thyroid disorders (1). Low iodine intake is associated with an increased risk of goiter, thyroid dysfunction, and developmental brain damage if the iodine deficiency is severe (1, 2). High iodine intake is associated with autoimmune thyroid disorder with an increased occurrence of hypothyroidism (3).

Iodine intake among populations in Greenland has been shown to be associated with the intake of traditional Greenlandic food items (4). The traditional Greenlandic diet includes iodine-rich seaweed (5), and previous studies have shown that intake of Greenlandic seaweed exceeded the iodine intake recommended by the WHO and a single meal caused a transient rise in serum TSH (6). It is recognized that the occurrence of thyroid autoimmunity and dysfunction increases with a rising iodine intake (7). Still, the importance of the dietary transition in Greenland for the occurrence of thyroid autoimmunity among Inuit in Greenland remains to be settled (8).

Greenland is Arctic environment and Greenland Inuit has developed a genetic susceptibility to metabolic disorders (9). This has been speculated to be due to adaptation to the lifestyle of Arctic habitat. It may be hypothesized that the intake of the iodine-rich traditional Inuit diet has caused an adaptation to high-normal iodine intake levels and hence an Inuit-specific association between the occurrence of thyroid dysfunction and iodine intake levels. This hypothesis was supported by our recent finding of a pattern of thyroid dysfunctions among iodine replete Inuit in Greenland comparable to those seen in populations with borderline iodine deficiency (10). It remains to be investigated if the Inuit-specific pattern of thyroid dysfunction is portrayed in thyroid autoimmunity. In addition, it remains to be settled if ethnic differences are present in thyroid autoimmunity.

Thus, in this study, we set out to describe the occurrence of thyroid autoimmunity among Inuit in Greenland and to assess the influence of different iodine intake levels on thyroid autoimmunity in the iodine-replete Inuit population of Greenland.

## Subjects and methods

### Area of investigations

In order to obtain the widest representation of the Greenlandic Inuit, Nuuk in West Greenland and Ammassalik district in East Greenland were selected for investigation.

Nuuk, the capital city of Greenland, is situated in West Greenland. It had 12,909 inhabitants at the time of investigation, of which 20% were non-Inuit. The lifestyle in Nuuk is more Westernized than the rest of the country and the diet consists mainly of imported produce.

Ammassalik district with a population of around 3000 inhabitants (95% Inuit) is located in East Greenland and is only accessible by plane. In the Ammassalik district, the town Tasiilaq and the settlements Tiniteqilaaq, Sermiligaaq, Kulusuk and Kuummiut were included, and settlements with less than 15 inhabitants were excluded for practical reasons.

Even though the urbanization has reached rural East Greenland, the lifestyle is less Westernized than on the west coast of Greenland. Hunting is still the main occupation and the diet consists predominantly of traditional diet, mainly from the sea (4).

### Subjects

We invited 1% of the Greenlandic population (*n*  = 561). A random selection of 225 inhabitants of Nuuk being 50–69 years old were invited and 211 attended. In Ammassalik district, we invited the whole population in the age group 50–69 years in Tasiilaq and the surrounding settlements (*n*  = 336). The participants represent a population that has shifted from the traditional way of living to a Westernized lifestyle in a matter of few decades. In sum, a total of 535 participants attended yielding an overall participation rate of 95%.

A participant was classified as Inuk (singular for Inuit) if the person and both parents were all born in Greenland. All other participants were classified as non-Inuit.

Non-Inuit were mainly skilled laborers from Denmark, who tend to move back to Denmark when they reach retirement and do not represent the general non-Inuit population. Hence, they were excluded from analysis of Inuit autoimmunity (*n*  = 101). In total, 434 Inuit participated, of which, 150 were from Nuuk in West Greenland and 284 were from Ammassalik district in East Greenland.

The Commission for Scientific Research in Greenland approved this study before the onset (reference no. 505-31). All subjects signed the informed consent form in participant’s chosen language (Greenlandic or Danish).

### Investigational procedures

A letter of invitation (in Greenlandic and Danish) was personally delivered to each subject by the local healthcare personnel. The invitations were delivered three times to non-responders before they were registered as non-attenders. The participants were examined at the local health care facility or, by request, at home visits. The participants were interviewed by one of the investigating physicians or by an interpreter, and the questions were asked as written in the questionnaires. The participants were asked about their dietary habits, lifestyle and medical history, including thyroid-related questions. Physical examinations on the participants were performed by the investigating physicians for major disabilities, examinations of the neck for visible goiter and measurements of height without shoes and weight in indoor clothing.

### Sample collection and assays

A non-fasting spot urine sample was collected in iodine-free polyethylene containers from the participants during the visit, and blood samples were drawn using minimal tourniquet. Serum was separated and samples were stored at –20°C until analysis. Blood sampling was missing in two participants and urinary iodine was missing in seven.

Iodine content in urine was determined by the Sandell–Kolthoff reaction modified after Wilson & van Zyl (11) as described in detail previously (12, 13). Urinary creatinine was determined by kinetic Jaffé method (14). Urinary iodine concentration varies with hydration and urine volume, and we thus adjusted excretion for dilution by calculating the iodine:creatinine ratio (4, 15), and this correction was done using the ethnic-specific creatinine excretion (16).

TPO-Ab was measured by RIA (DYNOtest^®^ anti-TPO, BRAHMS Diagnostica, Berlin, Germany), using native TPO from human thyroids as antigen. The TPOAb standards had been calibrated against the Medical Research Council (MRC) 66/387 with 1000 MRC units corresponding to 2800 BRAHMS units (manufacturer information) with a functional sensitivity of 30 U/mL for TPO-Ab (17). The TG antibodies (TGAb) were measured using Dynotest RIA (BRAH-MS Diagnostica) with a functional sensitivity of 20 kU/L for TGAb (18). The recommended cut-off for positive TgAb and TPOAb is 60 U/mL. We set the detection limit at the functional sensitivity, which was 30 U/mL for TPOAb and 20 U/mL for TgAb. Serum thyrotropin (TSH) was measured using LUMItest (BRAHMS, Berlin, Germany). The functional sensitivity of the TSH assay was 0.1 mIU/L and the reference intervals applied were 0.3–4.5 mIU/L based on the reference range settled for that assay (19). All assay runs included samples from different groups investigated in random order.

### Statistical analysis

Results are listed with numbers and percentages, and groups are compared using non-parametric statistics with Mann–Whitney *U* test for comparison of medians between two groups and chi-squared test for comparison of proportions. Explanatory variables entered in logistic regression models were sex, age (50–59 or 60–69 years), smoking habits (present, past or never smoker), alcohol intake (daily, occasionally or rarely) and iodine excretion (iodine between 100 and 200 µg/24 h, yes vs no; iodine between 100 and 300 µg/24 h, yes vs no). Hosting any thyroid antibody ((TPOAb and/or TgAb), TPOAb (with or without TgAb) or TgAb (with or without TPOAb)) was entered as dependent variables.

A *P* value of less than 0.05 was considered significant. Data were processed and analysed using Corel Quattro Pro 8 (Corel Corporation, Ottawa, Ontario, Canada) and the Statistical Package for the Social Sciences version 13.0 (SPSS Inc.).

## Results

Table 1 lists descriptive characteristics of participants.

**Table 1:** Descriptives of the participants from the capital city Nuuk in West Greenland and in town and settlements in rural Ammassalik district in East Greenland as reported in interview-based questionnaires.

	Inuit							
	Total		City		Town		Settlements	
	*n*	%	*n*	%	*n*	%	*n*	%
All Inuit	434	100.0	150	100.0	141	100.0	143	100.0
Gender								
Men	229	52.8	70	46.7	80	56.7	79	55.2
Women	205	47.2	80	53.3	61	43.3	64	44.8
Age								
50–59 years	259	59.7	87	58.0	87	61.7	85	59.4
60–69 years	175	40.3	63	42.0	54	38.3	58	40.6
Smoker^a^								
Present	328	75.8	111	74.5	108	76.6	109	76.2
Past	49	11.3	16	10.7	14	9.9	19	13.3
Never	56	12.9	22	14.8	19	13.5	15	10.5
Alcohol use^b^								
Daily	57	13.4	12	8.3	16	11.5	29	20.3
Occasionally	95	22.3	40	27.8	35	25.2	20	14.0
Rarely	274	64.3	92	63.9	88	63.3	94	65.7
Use of supplements with iodine	29	6.7	18	12.0	8	5.7	3	2.1
History of thyroid disease								
Hypothyroidism	4	0.9	1	0.7	2	1.4	1	0.7
Hyperthyroidism	5	1.2	1	0.7	2	1.4	2	2.1
Unknown	31	7.1	16	10.7	14	9.9	1	0.7
No	394	90.8	132	88.0	123	87.2	139	97.2
Goiter^c^	6	1.4	3	2.0	3	2.1	0	0.0
Previous treatment^d^								
Medicine	1	0.2	0	0.0	0	0.0	1	0.7
Radioiodine	1	0.2	1	0.7	0	0.0	0	0.0
Surgery	3	0.7	1	0.7	1	0.7	1	0.7
Thyroid medication at present	2	0.5	0	0.0	1	0.7	1	0.7
Thyroid disorder in 1st degree relative	8	1.8		6	4.0	1	0.7	0.7
Unknown	98	22.6	56	37.3	33	23.4	9	6.3
Visible goitre	1	0.2	1	0.7	0	0.0	0	0.0

^a^One non-Inuit missing; ^b^Nine missing; ^c^Data missing in one self-reported previous goitre; ^d^Data missing in two.

### Urinary iodine excretion

Mean estimated 24-h urinary iodine excretion was within the recommended range among Inuit in Nuuk (169 µg/24 h) and was more than adequate in East Greenland Inuit (Tasiilaq 224 µg/24 h; settlements 228 µg/24 h). Overall, urinary iodine excretion was <100 (*n*  = 102); 100–199 (*n*  = 135); 200–300 (*n*  = 81) and >300 µg/24 h (*n*  = 109).

### Thyroid dysfunction

TSH was above 3.6 mIU/L in 4.4% (*n*  = 19) of Inuit, while it was below 0.4 mIU/L in 6.7% (*n*  = 29) of Inuit, leaving 88.9% (*n*  = 384) with a TSH between 0.4 and 3.6 mIU/L.

### Thyroid autoimmunity

At least one of the thyroid autoantibodies measured was present in 8.3% (*n*  = 36) while not detected in 91.7% (*n*  = 396) of the participants. TPOAb and TgAb was present in 3.7% (*n*  = 16) and 5.9% (*n*  = 24), respectively.

Thyroid autoimmunity was more frequent among the Inuit in East Greenland compared to Inuit in West Greenland, as listed in Table 2 (*P*  = 0.02). The occurrence did not differ between the two age groups (Table 2).

**Table 2:** Prevalence rates of TPOAb and TGAb in Greenlandic Inuit cohort.

	Thyroid autoimmunity								*P*^a^
	TPOAb or TgAb		TPOAb		TgAb		TPOAb and TgAb		
	*n*	%	*n*	%	*n*	%	*n*	%	
East vs West Greenland									0.02
West Greenland Inuit	6	4.0	3	2.0	4	2.7	1	0.7	
East Greenland Inuit	30	10.6	13	4.6	20	7.0	3	1.1	
Age									0.86
50- to 59-year-olds	21	8.1	10	3.9	14	5.7	3	1.2	
60- to 69-year-olds	15	8.6	6	3.4	10	6.1	1	0.6	
Present smoking									0.08
Non-smoker	13	12.5	7	6.7	8	8.2	2	1.9	
Smoker	23	7.0	9	2.8	16	5.2	2	0.6	
Alcohol consumption in 2 groups									0.29
Less than 7 units per week	26	9.6	14	5.1	15	5.7	3	1.1	
More than 7 units per week	10	6.6	2	1.3	9	6.4	1	0.7	

^a^Chi-squared test for comparing the differences in the prevalence of TPOAb or TGAb in subgroups.

**Figure 1:**
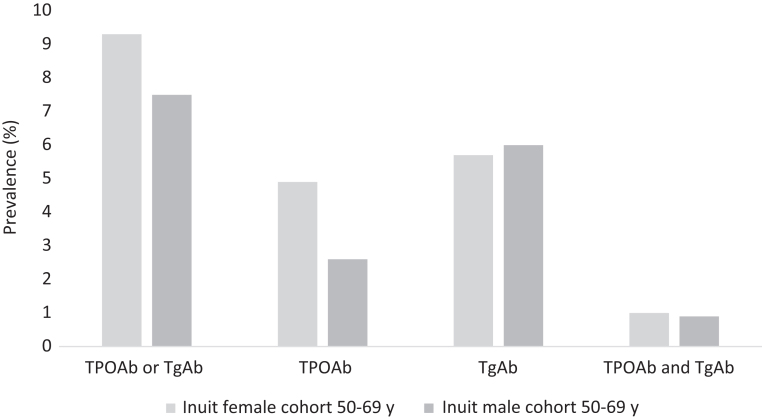
Prevalence of thyroid autoimmunity in Inuit in Greenland by gender.

TPOAb or TgAb was found in 9.3% (*n*  = 19) and 7.5% (*n*  = 17) in the Inuit women and men, respectively, with individual values for TPOAb, TgAb and harbouring both TPOAb and TgAb visualized in Fig. 1. Thus, there was no difference between men and women in the occurrence of thyroid autoantibodies (*P*  = 0.50).

There was a non-significant tendency suggesting that thyroid autoimmunity was more frequent among non-smokers (12.5%) compared to smokers (7.0%) (*P*  = 0.08), and a similar non-significant trend was seen with alcohol consumption (*P*  = 0.29) (Table 2).

**Figure 2:**
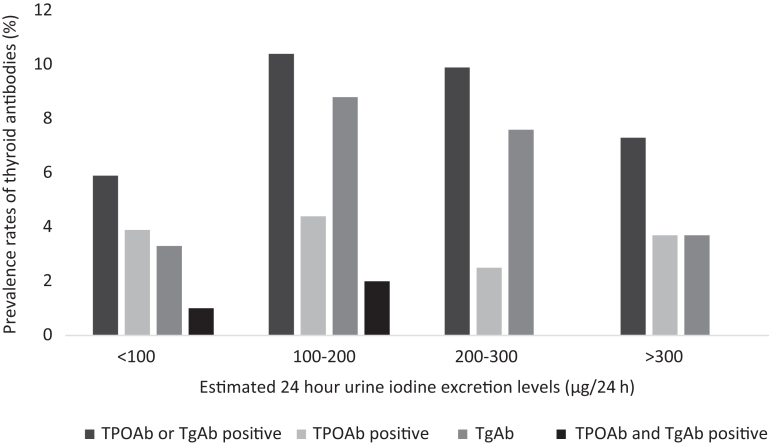
The prevalence of thyroid antibodies with four different iodine excretion levels among Greenlandic Inuit in Nuuk, West Greenland and Ammassalik district, East Greenland.

There was no statistically significant difference in the prevalence of thyroid autoantibodies between participants with the recommended iodine intake level when compared to those with iodine intake below or above the levels recommended by WHO (*P*  = 0.83), as shown in Fig. 2.

**Figure 3:**
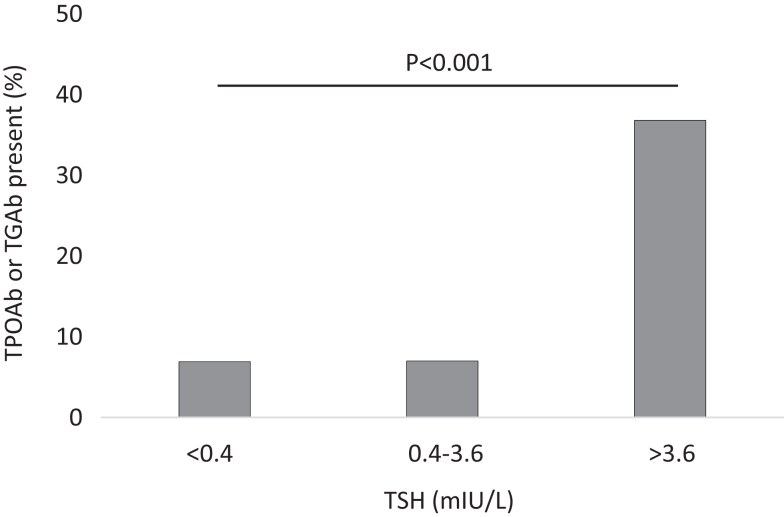
Prevalence of thyroid autoantibodies in Inuit in Greenland by TSH levels.

We found strong evidence that median TSH was higher among participants with the presence of any thyroid autoantibody (*P*  = 0.005), primarily related to TPOAb (*P*  = 0.001) while not TgAb (*P*  = 0.10). Conversely, harbouring any thyroid autoantibody was most frequent in participants with TSH above 3.6 mIU/L: any thyroid antibody, *P*  < 0.001; TPOAb, *P*  < 0.001; TgAb, *P*  = 0.001. This is illustrated in Fig. 3.

The occurrence of TPOAb and/or TgAb or TPOAb alone was not influenced by iodine, smoking, alcohol, sex or age group when tested in logistic regression models. However, participants with urinary iodine excretion between 100 and 300 µg/24 h were more likely to host TgAb (odds ratio: 2.5; 95% CI: 1.03–6.2; *P*  = 0.04) compared to participants with urinary iodine excretion below 100 or above 300 µg/24 h.

## Discussion

This is the first study of thyroid autoimmunity in Inuit. We found a substantially lower occurrence of thyroid autoantibodies than expected from the high iodine intake levels. The occurrence of thyroid autoantibodies was 8.3%, and the prevalence was higher among Inuit in the rural Ammassalik district with the highest iodine intake compared to the Inuit in the capital city Nuuk. Interestingly, we found a limited difference between women and men and between smokers and non-smokers.

Autoantibodies against the thyroid gland have been demonstrated in serum in population studies with TPOAb and TgAb being the most commonly occurring antibodies (17, 20). They are frequent in many populations (21), but data were lacking for Inuit. In a study by Li *et al.* performed in three areas in China with different iodine intake, the prevalence of TPOAb or TgAb in an age group parallel to ours was around 15% of women and 7% of men with more than adequate iodine intake (22). With excessive iodine intake, these numbers were around 20 and 12%, respectively (22). In a comparative study by Laurberg *et al.* carried out in Iceland with more than adequate iodine intake, and Jutland, and Denmark, with mild and moderate iodine intake before iodine fortification of salt, the occurrence of TPOAb or TgAb was around 20% in women and 10% in men with more than adequate iodine intake (23). When the iodine intake level was in the range of deficiency, these numbers were 35 and 20%, respectively (23), and in a parallel population with mild and moderate iodine, the prevalence was 30% in women and 11% in men (17). All of these numbers are markedly above the relatively rare occurrence in Inuit.

Our population in West Greenland was iodine replete, while the participants in East Greenland had an iodine excretion above the recommended range, and the difference in the occurrence of thyroid dysfunctions conforms to these levels with the highest prevalence of hypothyroidism in East Greenland (10). They are similar to those reported in other studies of thyroid autoimmunity (22, 23) suggesting an influence of iodine nutrition on thyroid autoimmunity. However, Bulow *et al.* found an increased occurrence of thyroid autoimmunity following a raised iodine nutrition level among younger individuals only (24), whereas the occurrence of thyroid antibodies was unaltered in subjects aged above 60 years. This conforms to our finding suggesting that iodine intake had limited influence on the occurrence of thyroid autoimmunity in the age group included in our study.

Age-associated decline in the immune function is accompanied by higher levels of inflammatory markers (25) in accordance with replicative senescence with decreasing environmental influence. Hence, the low level of thyroid autoantibodies in our study was unlikely due to the age range of the participants surveyed.

Smoking is associated with lower occurrence of TPOAb or TgAb. The prevalence of either TPOAb or TgAb was around 20% among non-smokers and 15% among smokers in the DanThyr study (26) with a clearer trend for TgAb (15 vs 7%) than for TPOAb (15 vs 12%). The negative association between smoking and the presence of thyroid autoantibodies in serum was also shown in the NHANES III study with thyroid autoantibodies in 18% of non-smokers and 11% of smokers (27). Smoking was frequent among Inuit with three out of four being present smokers, and a trend towards a difference in the occurrence of TPOAb and TgAb between smokers (7%) and non-smokers (12.5%) was not statistically significant (*P*  = 0.08). Still, the frequency was only half of that seen in other populations among both smokers and non-smokers, and smoking does not explain the markedly lower occurrence of thyroid autoantibodies among Inuit. Thus, the number of smokers in our data was similar between Inuit in East and West Greenland, while the prevalence of thyroid autoantibodies differed. This suggests that ethnicity plays a role. The NHANES III study reported an influence of ethnicity on the effect of smoking on the occurrence of thyroid antibodies with an attenuated odds ratio in non-Hispanic blacks compared with the other ethnic groups (27). This is in keeping with our data suggesting an influence of ethnicity.

The importance of genetic factors was suggested from information on relatives to patients with autoimmune thyroid disease (28), and details on the genetic susceptibility were provided by the study of twins (29). This study reported a genetic influence on serum TPOAb of around 70% in women and 60% in men and for TgAb the influence was around 75 and 40%, respectively. Our participants comprised a distinct ethnic group (30) with a high iodine intake, and our finding of a low occurrence of TPOAb and TgAb compared to the findings in parallel populations is in keeping with a genetic influence.

The limited sex difference with the presence of a thyroid autoantibody among 9.3% of Inuit women and 7.5% of Inuit men differs from the pattern in other populations with a prevalence of any thyroid antibodies being two to four times higher in women compared to men (17, 22, 31). This dampened difference in Inuit could relate to a genetic component in the occurrence of thyroid autoimmunity with Inuit being a distinct ethnic group. It could thus be speculated if further studies into autoimmunity in Inuit may provide a clue to the understanding of a genetic component in thyroid autoimmunity.

An interesting path to consider is the potential modulation of the immune responses by thyroid hormones (32) influenced by T3 levels altered by the cold that comes with Arctic residence (33). Furthermore, selenium may influence thyroid autoimmunity. Data suggest an increased prevalence of thyroid autoimmunity among individuals with selenium levels in the blood below 70 µg/L (34). Inuit exhibited distinctly higher selenium status in previous studies because of their frequent intake of marine mammal food (35, 36). The high selenium levels may have a protective effect on thyroid autoimmunity despite the high levels of iodine. Finally, the potential influence of persistent organic pollutants common in Greenland Inuit (37) should be considered in future evaluations as they are a health concern in relation to the thyroid (38).

The number of participants was a limitation to our study and may have weakened the strength of evidence of differences between groups. Hence, the statistically insignificant trends found in our data might have shown significant differences if the number of participants with thyroid autoantibodies had been higher. However, we included 1% of the total population of Greenland and the high participation rate supports the validity of our findings. Still, our results should be interpreted with these numbers in mind and remembering that the Inuit population globally is limited in numbers.

In conclusion, these first data on thyroid autoimmunity among Inuit show a very low occurrence of thyroid autoantibodies among Inuit in Greenland. Our findings are consistent with the previously reported low prevalence of hypothyroidism in the Greenlandic Inuit, and they support the speculations on a genetic adaptation to iodine excess among Inuit with iodine abundancy for centuries. East–West differences conform to iodine nutrition while the lack of differences with smoking and sex further support thyroid autoimmunity among Inuit to stand out. The low frequency and details on the influence of iodine are unique to Inuit and warrant further surveys to describe both genetic factors and the influence of environmental factors such as cold, selenium and persistent organic pollutants.

## Declaration of interest

The authors declare that there is no conflict of interest that could be perceived as prejudicing the impartiality of the research reported.

## Funding

The study was supported by grants from the Government of Greenland and Karen Elise Jensen Fond.

## Data availability

Restrictions apply to the availability of data to preserve patient confidentiality. The corresponding author will on request detail the restrictions and any conditions under which access to some data may be provided.

## Author contribution statement

Paneeraq Noahsen: formal analysis, writing – original, draft, visualization. Karsten F Rex: data curation, writing – review and editing. Inge Bülow Pedersen: Formal analysis, writing – review and editing. Gert Mulvad: Resources, investigation. Hans Christian Florian-Sørensen: Resources, investigation, Michael Lynge Pedersen: Resources, investigation, writing – review and editing. Stig Andersen: Conceptualization, methodology, formal analysis, investigation, resources, data curation, supervision, project administration, funding acquisition, writing – review and editing.
